# Relationship of Resilience Factors With Biopsychosocial Markers Using a Comprehensive Home Evaluation Kit for Depression and Suicide Risk: A Real-World Data Analysis

**DOI:** 10.3389/fpsyt.2022.847498

**Published:** 2022-05-30

**Authors:** Sooah Jang, Sun-Woo Choi, Ryunsup Ahn, Ju-Yeal Lee, Joohan Kim, Jeong-Ho Seok

**Affiliations:** ^1^Research Institute of Minds AI, Co. Ltd., Seoul, South Korea; ^2^Institue of Behavioral Sciences in Medicine, College of Medicine, Yonsei University, Seoul, South Korea; ^3^Department of Communications, Yonsei University, Seoul, South Korea; ^4^Department of Psychiatry, Gangnam Severance Hospital, Yonsei University College of Medicine, Seoul, South Korea

**Keywords:** resilience, mentalization, hypothalamic-pituitary-adrenal axis (HPA axis), salivary cortisol, adverse childhood experience (ACE), depression

## Abstract

**Objectives:**

Adverse childhood experiences (ACEs) are fundamental factors in developing depression with increased suicide risk. Resilience is considered an important protective factor that can prevent trauma survivors from developing depression. We developed a home evaluation kit for a comprehensive assessment of bio-psycho-social factors related to depression and suicide. This kit contained a psycho-social evaluation battery, named the Protective and Vulnerable factors battery questionnaire (PROVE) comprising depressive symptoms and suicide risk, as well as various depression-related psychosocial factors, such as ACE, resilience, mentalization capacity, and attachment, *via* online survey tools. Furthermore, salivary cortisol levels were used as biological indicators to assess the hypothalamus-pituitary-adrenal axis function.

**Methods:**

Real-world data analysis was made out of data collected from participants who visited CHEEU Counseling center or Gangnam Severance hospital for mental health check-ups. The participants were put into three mental state groups (green-normal, yellow-borderline, and red-risk) depending on the result of PROVE battery. The difference between psychosocial factors and salivary cortisol indicators by the group was identified by analysis of covariance with sex and age as covariates. Linear regression analysis was conducted to find a significant association of resilience score with other bio-psycho-social variables, such as ACE, attachment, mentalization, or post-awakening cortisol concentrations (area under the curve with respect to ground, AUCg). A partial correlation analysis was performed to evaluate the relationship of AUCg with psychosocial factors.

**Results:**

Depression-related psycho-social indicators were significantly different among groups. Insecure attachment and the mentalization problem are negatively influencing factors to resilience. Furthermore, the severity of depression in participants with ACE was also influenced by mentalization problems. AUCg was different according to the PROVE group, presence of ACE, or resilience level. In addition, AUCg showed a positive correlation with resilience score but negative correlations with depressive symptoms, ACE, mentalization problems, and anxiety or avoidance attachment.

**Conclusion:**

This study suggests that there are some key factors negatively affecting resilience: insecure attachment and mentalization problems. In groups with ACE, a mentalization problem was suggested as a factor that can increase depressive symptoms. AUCg was associated with resilience as well as several other vulnerable factors of depression, showing its potential as a promising biomarker.

## Introduction

Depression, one of the leading contributors to the global disease burden, has various causative factors and development pathways (1). Adverse childhood experience (ACE) is a well-studied predisposing factor for severe and chronic depression, which is related to elevated suicidal risk (2, 3). ACE includes various types of childhood maltreatment, such as physical, emotional, and sexual abuse, and neglect (4). Recently, the concept of ACE has been extended to include multifaceted stressful childhood experiences, including exposure to domestic violence and bullying (5, 6). ACE affects brain development through various pathways and makes it difficult for an individual to cope with stressful conditions. Brain regions, such as the amygdala, hippocampus, and prefrontal cortex, and neural circuits, such as the hypothalamic-pituitary-adrenal (HPA) axis are particularly affected, compromising their ability to process emotionally-laden or neutral stimuli (7, 8). Eventually, these changes force the brain more predisposed to depression (2, 3).

In particular, the HPA axis is a main neurobiological mechanism that mediates ACEs to cause stress-related disorders, including depression (9). Repetitive or frequent stressors have cumulative effects on allostatic load in the long term, which is linked to alterations in the function of the HPA axis (10). Multiple evidence showed that early adversity may impact the reactivity and regulation of the HPA axis (10). Chronic stressors initially exhibit elevations in cortisol, but often lead to blunted reactivity, which can co-occur with hyper-responsiveness of other stress systems (11). These abnormalities of the HPA axis and stress response system in the depressive disorder have been implied in several hundred studies (12). A recent study identified that lower cortisol levels explained between 10 and 20% of the total associations of ACE with depressive symptoms (9). Taken together, ACE induces a vulnerable state of depression through these changes.

Several studies have shown that 30–40% of children do not develop psychiatric disorders even after being the victims of ACEs (13–15). The main protective factor that prevents depression in these trauma survivors is called resilience, which is the ability to set of adaptive characteristics enabling an individual to cope with and recover from stress or trauma (16). Resilient people who are capable of focusing on positive aspects, establishing healthy relationships with others, and are emotionally conscious and optimistic (17). Promoting resilience can improve physical and mental health, prevent depression, and facilitate favorable treatment outcomes in case trauma survivors develop depression (17). Therefore, it is important to understand what factors could contribute to improving the resilience of patients with ACEs. For the proper evaluation and enhancement of resilience, it is necessary to understand the constructs of resilience and the mechanism that cultivates resilience. We focused on three factors that have been known to be related to resilience: cognitive appraisal, attachment, and stress-response system (18–21).

From the perspective of the positive appraisal style theory of resilience (PASTOR), the discussion of various levels surrounding resilience-from socioeconomic to genetic-has been integrated to some extent (22). According to the PASTOR, a positive cognitive appraisal (evaluation) mechanism protects against stressful life events (22). Here, potentially stressful stimuli are processed internally (appraisal) using various cognitive functions, and the result determines the emotional response to those stimuli. The appropriate functioning of higher-order cognition employed during such processing largely depends on appropriate judgments of social contexts (23). Following underpinning appraisal mechanisms could be crucially determinant of resilience: (1) positive situation classification (which means positively interpreting the current situation) (2) retrospective positive reappraisal of threat (which generate second-order representations of mental states to mitigate negative appraisals in aversive situations) and (3) inhibition of retraumatizing triggers (22). All these cognitive mechanisms are closely related to mentalization, which is necessary for flexible appraisal in the social context (24, 25). Mentalization is the ability to understand the mental state of oneself or others. This is central to a mutual understanding of relationships, self-control, and flexible perception and judgment of the world around in a social context (26). The balanced mentalization is developed through empathic interaction between the child and attentive caregivers and indicates a necessity for secure attachment (27). The mentalization capacity is developed by mirroring in infancy and demonstrating a mentalization narrative about the surrounding world of the child by the caregiver (28). Therefore, children with secure attachment show better mentalizing capacity; they can interpret the world around them properly, and develop positive cognitive appraisal mechanisms, which is crucial to cope with stress more resiliently (21, 26). On the other hand, the absence of secure attachments in early life can lead to distorted mentalization and problems in cultivating resilience (29, 30). Hence, attachment has been shown to be an influential factor for psychological adjustment in individuals who have experienced various kinds of trauma (30, 31). Victims of early life stress need to be supported to promote constructs of resilience and develop protective factors against depression.

On the other side, there have been attempts to find biomarkers of resilience in individuals with ACEs. The dysregulation of the stress response system (the HPA axis) in individuals with ACEs has been focused on as another important mediator of resilience (32, 33). The HPA axis dysfunction can be assessed by measuring cortisol indicators, such as post-awakening cortisol concentrations (area under the curve with respect to ground, AUCg) or cortisol awakening response (CAR or area under the curve with respect to increase, AUCi). Several studies have reported that high resilience is associated with elevated AUCg or AUCi, and these indices can be considered potential biomarkers (34–36). Since most resilience evaluations are assessed using a self-reported scale, the addition of an objective biomarker can help achieve a more scientific evaluation of protective factors.

To sum up, considering resilience in depression evaluation, it is necessary to comprehensively assess several parameters, such as vulnerability (early-life stressors and blunted HPA axis) and protective factors (resilience, secure attachment, and mentalization capacity), as well as symptoms severity. However, there are limited studies that investigated these parameters in depression patients through the integrated bio-psycho-social evaluation. Few review articles suggested that depression should be understood upon the stress-diathesis models (37, 38). In addition, Iob et al. (9) comprehensively clarified the relationship between depressive symptoms and salivary cortisol levels in people with ACE and Laurent et al. (39) explored bio-psychological parameters related to resilience. These studies suggest the possibility that ACEs influence the biological mechanism called the HPA axis and a psychological factor called mentalization and may affect the development of resilience and subsequent depressive symptoms. These previous study results led us to develop an evaluation kit, Minds.NAVI^TM^ enables an inclusive assessment of biopsychosocial factors of depression and suicide risk for patients with depressive disorder.

The aim of this study is to investigate the following: (1) factors that influence resilience in the entire group and in each group by depression risk; (2) protective factors against depression in people with ACEs through bio-psycho-social evaluation with Minds.NAVI^TM^; (3) clarify the group differences in salivary cortisol indicators (AUCg and AUCi) and the association of AUCg with psycho-social factors to investigate the possibilities of AUCg as a biomarker of depression within the context of resilience. We hypothesized that secure attachment, high mentalization capacity, and high post-awakening cortisol concentrations would serve as factors that enhance resilience in the entire group, each risk-level group, and also in the group with ACEs (21, 36). It was also assumed that the same factors would be related to decreasing depressive symptoms in people with ACEs (30, 40, 41). Furthermore, AUCg was expected to be highly correlated with the resilience and the psychosocial factor associated with it and making it a potential biomarker (34–36).

## Methods

### Participants

**Figure 1:**
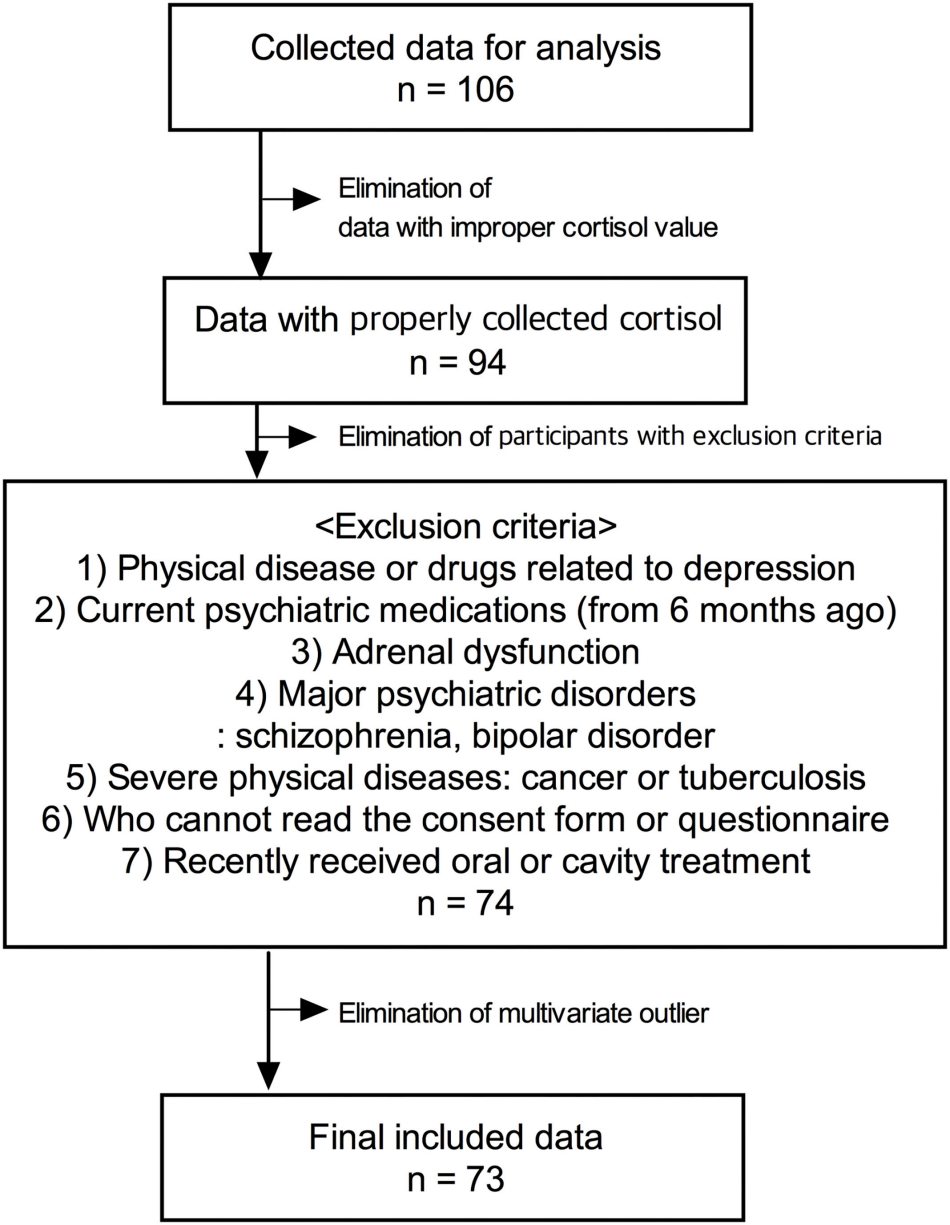
Flow chart summary of the participation exclusion process for analysis.

Data were collected from participants who visited the CHEEU Counseling Center and Gangnam Severance Hospital and participated in the pilot mental health evaluation project of Minds AI Co. Ltd using Minds.NAVI^TM^. The data were not gathered by dividing the disease group and control group through the design for clinical trials, and data of participants who received mental health evaluation were analyzed retrospectively. Among the participants, there were patients with psychiatric histories or taking drugs, and there were healthy adults. The exclusion criteria were as follows: (1) subjects who have physical diseases or taking drugs that can cause depression; (2) subjects who have been on psychiatric medications for the last 6 months; (3) subjects who have adrenal dysfunction; (4) subjects who have been diagnosed with major psychiatric disorders, such as schizophrenia or bipolar disorder; (5) patients with severe physical diseases, such as cancer or tuberculosis; (6) foreigners and illiterates who cannot read the consent form or questionnaire; (7) subjects who recently received oral or cavity treatment. The final analysis was conducted using the data of a total of 73 participants with an exclusion process (Figure 1). The protocol used for this retrospective study was reviewed by the Institutional Review Board of Gangnam Severance Hospital, Yonsei University College of Medicine [No.3-2021-0451].

### Assessment–Minds.NAVI^TM^

The mental health evaluation was conducted using Minds.NAVI^TM^, which was developed by Minds AI Co. Ltd. (Seoul, Republic of Korea). Minds.NAVI^TM^ consists of a self-report questionnaire about protective and vulnerable factors related to depression and salivary hormone analysis (42).

#### PROtective and Vulnerable Factors BattEry Questionnaire

A self-report questionnaire is a battery tool for the screening and assessment of depression, the PROtective and Vulnerable factors battEry (PROVE). The PROVE test consists of 6 subdomains of depressive symptoms (PROVE-DS), suicide risk (PROVE-SR), adult attachment type (PROVE-AAT), ACE (PROVE-ACE), mentalization capacity problem (PROVE-MC), and resilience (PROVE-KRQ). It is designed to comprehensively evaluate not only depressive symptoms but also protective-vulnerable factors that can affect the overall disease course and treatment plan. The validity and reliability of the PROVE test were verified by comparative analysis with widely used standardized scales (42).

##### PROVE-DS-Depressive Symptomatology Section

This subdomain consists of a 0–4 Likert 5-point scale of a total of 15 questions. They are asked to review their status for the past 2 weeks and respond to the degree of symptoms related to depression. Zero to 8, 9 to 25, 26 to 37, 38 to 45, and 46 to 48 points indicate no, minimal, mild, moderate, and severe depression, respectively. The Cronbach's α of PROVE-DS was 0.93.

##### PROVE-SR-Suicide Risk Section

This is a subdomain to evaluate suicide ideation and risk and includes six questions. Five are yes/no questions and 6th is a 1-4 Likert 4-point item. The total score ranges from 0 to 20, 0 to 4, 5 to 7, and 8 or more indicating low, borderline, and high suicide risk, respectively.

##### PROVE-AAT-Adult Attachment Type Section

This is a subdomain to investigate adult attachment, and the responses are based on the intimate and close relationships that the participants currently have. It consists of attachment anxiety and avoidance subscales. Attachment anxiety assesses the degree to which an individual is preoccupied with an attachment object or fears rejection/abandonment, while attachment avoidance is a level of reluctance and discomfort in getting close to the other person. It includes nine questions each about attachment anxiety and avoidance on the Likert 7-point scale and some items are reversely scored. The total range of each subscale is 9 to 63 points. A higher score indicates more anxiety or avoidance. Based on the two subscales, adult attachment types are finally classified into the following four types: (1) secure, low attachment anxiety and avoidance scores, (2) dismissing, low attachment anxiety score and high attachment avoidance score, (3) preoccupied, high attachment anxiety score and low attachment avoidance score, (4) disorganized, high attachment anxiety and avoidance scores. The Cronbach's α of attachment anxiety and avoidance score were 0.93 and 0.77, respectively.

##### PROVE-ACE-Adverse Childhood Experience Section

This subdomain evaluates negative experiences, such as abuse, neglect, and bullying during early life. It contains a total of 52 items on a 4-point Likert scale and 6 subscales, and measures (1) emotional abuse (5 items), (2) physical abuse (9 items), (3) sexual abuse (10 items), (4) neglect (10 items), (5) exposure to domestic violence (10 items), and (6) bullying (8 items) during childhood or adolescence. A higher score indicates more negative experiences during early life. The cutoff score for each subscale differs according to sex. In this study, participants were classified as having or not having ACE depending on whether any item exceeded the cutoff of each subscale or not. The Cronbach's α of PROVE-ACE was found to be 0.95 and by sub-scale, 0.86 for emotional abuse, 0.88 for physical abuse, 0.92 for sexual abuse, 0.9 for neglect, 0.93 for domestic violence, and 0.9 for bullying.

##### PROVE-MC-Mentalization Capacity Problem Section

This is a subdomain to identify mentalization problems and consists of five sub-factors, including a total of 16 items with a 5-point Likert scale. Five sub-factors are as follows: (1) lack of emotional awareness (4 items, cutoff score 10), (2) lack of emotional expression and interaction (4 items, cut-off score 10), (3) psychic equivalence mode (2 items, cutoff score 6), (4) hasty incomplete mentalizing (3 items, cutoff score 9), and (5) lack of mentalizing others (3 items, cutoff score 5). A higher score indicates a failure of the mentalizing process and a lack of mentalization capacity. The Cronbach's α of sub-factors of PROVE-MC was 0.47–0.76.

##### PROVE-KRQ-Resilience Section

The Korean Resilience Quotient (KRQ)-53, modified and supplemented Resilience Quotient Test (RQT) developed by Reivich and Shatte according to the Korean situation, was used to measure resilience in PROVE battery (21, 43, 44). Resilience can be classified into three sub-factors as self-regulation ability, interpersonal relationship ability, and psychological optimism. KRQ is a 5-point Likert-type scale consisting of 53 items, and a high score can be interpreted as highly resilient. A total score of 212 or higher, and 181–211, and 180 or less are classified as high, average, and low resilience, respectively. The Cronbach's α of PROVE-KRQ was found to be 0.92 and by sub-scale, 0.83 for self-regulation ability, 0.85 for interpersonal relationship ability, and 0.89 for psychological optimism.

##### Group Classification Algorithm

**Figure 2:**
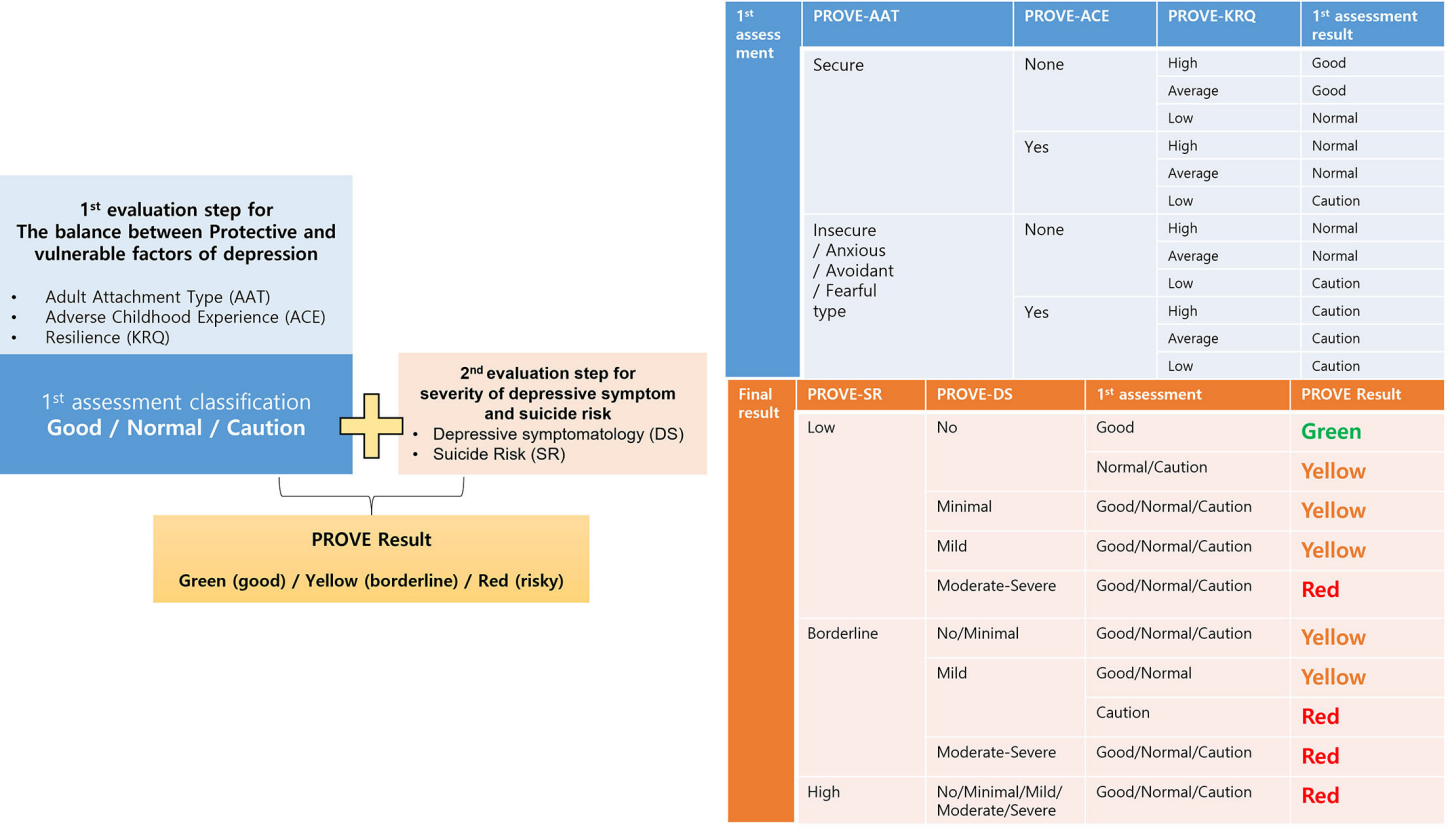
Flow chart summary of the analysis of results of the PROtective and Vulnerable factors battEry questionnaire (PROVE) battery. The first assessment is based on protective and vulnerable factors of depression, and these results are combined with the current depression, and finally, the participants are divided into the following three groups: good (green) /borderline (yellow)/risky (red). The tables next to it describe the detailed logic from which each process is derived. PROVE, PROtective and Vulnerable factors battEry questionnaire; AAT, adult attachment type; ACE, adverse childhood experience; KRQ, Korean resilience quotient; DS, depressive symptomatology; SR, suicide risk.

The comprehensive PROVE-battery results are presented as green-healthy, yellow-borderline, or red-risky mental health states by integrating the results of the first and second evaluation steps (Figure 2). The first evaluation step includes results of PROVE-AAT, PROVE-ACE, and PROVE-KRQ, which may have an impact on the development of depression. Based on the results of these three subdomains, the balance between the protective and vulnerable factors for depression can be categorized into “good,” “normal,” or “cautious” conditions (Figure 2). If a participant has no vulnerability factor regarding insecure attachment, positive ACE history, and deficient resilience, one's balance is categorized as “good.” When he/she has one vulnerable factor among three subdomains, one's balance is categorized as “normal.” If there are two or more vulnerable factors reported, this balance is considered “cautious.” In the second evaluation step, the groups were divided into depressive or non-depressive subgroups with/without suicidal risk based on the severity of PROVE-DS and SR. Considering the results of the first evaluation result together, the final PROVE battery result is presented as a green-healthy, yellow-borderline, or red-risky mental health state. If the protective-vulnerable factor balance is not “good” but “normal,” one may be categorized as a yellow group because there is a risk of depression even without significant depressive symptoms or suicidal risk (41, 45, 46). Otherwise, if a person is categorized as a “cautious” subgroup and reports minimal or mild depressive symptoms in the second assessment step, one can be finally classified as a “red” state. The classification algorithm for PROVE is presented in Figure 2.

#### Salivary Cortisol Analysis

Saliva samples were collected to examine the HPA axis function indexed by the post-awakening cortisol concentrations (AUCg) and CAR (AUCi). The AUCg is the total cortisol secretions during the post-awakening period and was calculated as the area under the curve with respect to the ground from the time point immediately after awakening to 60 min (47). The CAR, i.e., the value to be referred to in this article as AUCi, is characterized by an increase in cortisol levels within the first 30 min after waking up in the morning (48, 49). Both AUCg and AUCi are reliable indices of the HPA axis function (47). Although there was a lack of measurement of an hour of awakening time, we tried to make it up by offering the first cortisol sample on awakening (S1). Participants were instructed to collect their saliva samples immediately upon awakening (0 min), and 30 and 60 min after awakening on a day with the usual level of stress. Saliva was collected without external stimulation, but with muscle movement and expectoration into the collection tube (Simport Inc., QC, Canada) with a minimum volume of 2 ml at each time point. The participants were asked to not smoke, eat, drink, or brush their teeth for 30 min before the saliva collection. Collected samples were centrifuged (10,000 x *g* for 15 min at 4°C) to precipitate mucins and debris, and the supernatants were collected and stored at−70°C until further analysis. For the precise interpretation of salivary cortisol data, there are many things to consider, such as time of awakening, sleep quality, sea, age, sex, socioeconomic status, and body mass index, but we only corrected age and sex due to insufficient information.

##### Exclusion of Salivary Cortisol Data Collected From Non-Adherent Participants

The typical CAR is defined as an increase in cortisol levels to at least 2.5 nmol/L above baseline (i.e., CARi > 2.5 nmol/L) in healthy participants (50). Non-compliance (first saliva sample collection after a delay of more than 10 min after awakening) is known to be a primary cause of failure to capture the typical CAR in healthy participants (51). In preliminary assays, few participants (*n* = 12) showed no typical CAR, and the cortisol concentrations at waking, and 30, and 60 min after waking were 14.1 ± 12, 12.4 ± 5.1, and 19.4 ± 6.8 nmol/L, respectively; non-compliance data were removed from the further analyses. The data obtained from 73 compliant participants (mean age, 33.9 years; range, 35 years) were moved to the final analysis in this study.

##### Measurement of Salivary Cortisol

The cortisol levels in saliva were measured by radioimmunoassay (RIA) as previously described (52, 53). Cortisol antisera were purchased from Accurate Chemical & Scientific Co. (NY, USA). Cortisol antiserum cross-reacts with cortisone, 11-deoxycortisol, prednisolone, cortisol-21-glucosiduronate, cortisol-21-sulfate, and other steroids with cross-reaction levels of 1, 8.9, 31.6, 1.3, <0.01, and <0.01%, respectively. Standards, quality control materials, and saliva samples were routinely assayed in duplicate. The interassay coefficients of variation (CVs) as assessed by quality controls with mean cortisol concentrations of 3.6 and 10.9 nmol/L were 7.4 and 8.5%, respectively (*n* = 1). The analytical sensitivity for cortisol was 0.4 nmol/L.

### Statistical Analysis

Since the cortisol values did not show normal distribution, we used this value by putting a square root on each cortisol value (54). In analysis, the hour of awakening time was not included due to lack of measurement (47, 49). Two methods were used to prove the legitimacy of the current cortisol sampling time. The first one is to remove the sample showing the explicitly atypical pattern according to the described above, and the second one is to present that there is no group-specific difference of S1 (47, 49). Differences in demographic characteristics among PROVE-battery groups were compared using an independent sample ANOVA for numerical variables or a Chi-square (χ^2^) test for categorical variables. All of the following analyzes have entered the sex and age as covariates. Analysis of covariance (ANCOVA) was used to compare differences in psychological indicators or salivary hormones among different groups. Univariate and multivariate regression analyses were used to explore indicators related to resilience or depression severity. Multicollinearity was not observed, because all variance inflation factor (VIF) values were <1.5. The association between cortisol hormone and psychological indicators was investigated with a partial Pearson correlation analysis. The statistical threshold was set at *p* < 0.05. All statistical analyses were conducted using IBM SPSS version 25 (IBM Corporation, Armonk, NY, USA). Identifying multivariate outliers using a robust variant of the Mahalanobis distance was done and 1 case was found to be an outlier and excluded from the final analysis.

## Results

### Demographic and Psychological Characteristics of Each Group

**Figure 3:**
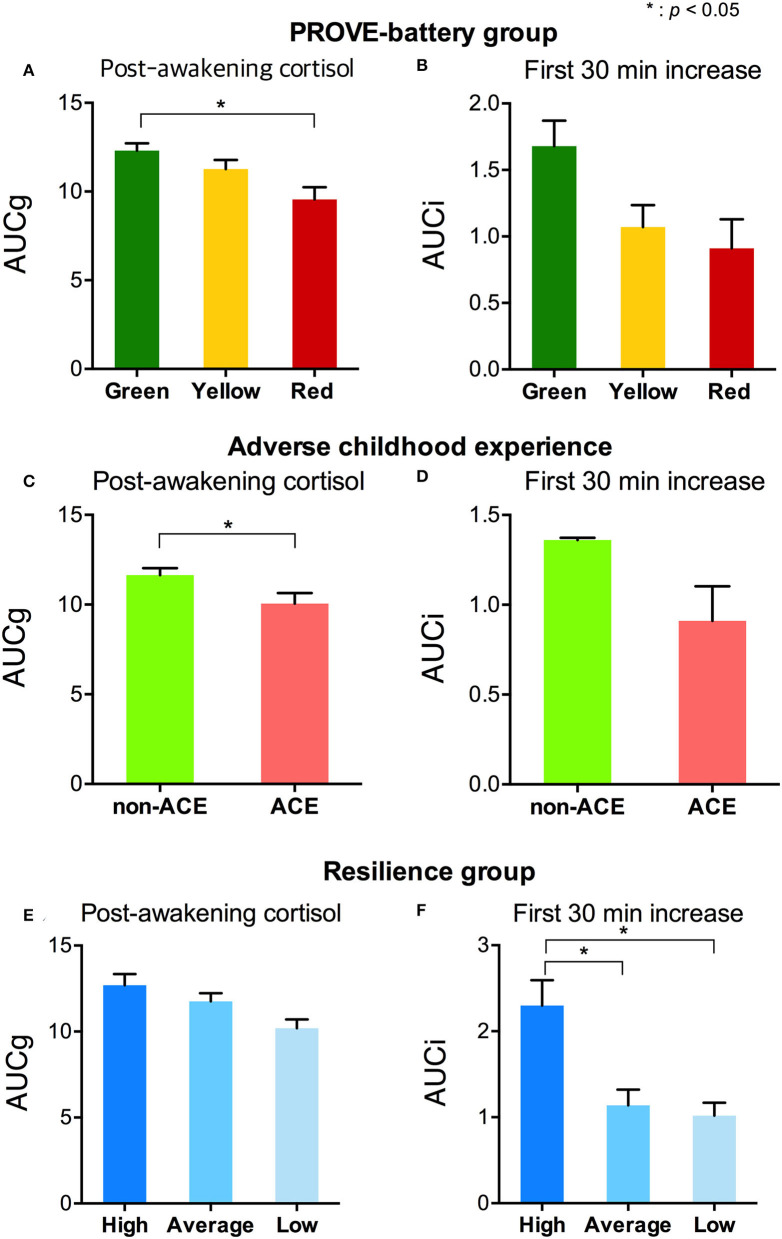
Stress-related salivary cortisol indicators by the group with PROVE battery, presence of ACE, and resilience group. **(A)** AUCg and **(B)** AUCi of each PROVE-battery group were represented. **(C)** AUCg and **(D)** AUCi were shown according to the presence or absence of an adverse childhood experience. Differences of **(E)** AUCg and **(F)** AUCi by each level of resilience were presented. PROVE, PROtective and Vulnerable factors battEry questionnaire; ACE, adverse childhood experience; AUCg, area under the curve with respect to the ground of post-awakening cortisol concentrations; AUCi, area under the curve with respect to the increase of cortisol awakening response. *p < 0.05.

Figure 1 represents the participants' exclusion process. Table 1 shows the demographic characteristics of the study participants of PROVE battery groups. The age of the participants was between 21 and 58 years old. There were significant differences in age, sex, and psychiatric disorder history among PROVE groups. The participants in the healthy (green) group were older than those of the other groups (green vs. red, *p* = *0.0*07; green vs. yellow, *p* = 0.045). The risky (red) group had a higher proportion of female participants as compared to the other two groups (red vs. green, *X*^2^ = 4.083, *p* = 0.043; red vs. yellow, ***X***^**2**^ = 10.544, *p* = 0.001) and more participants with psychiatric disease history than the green group (red vs. green, ***X***^**2**^ = 8.32, *p* < 0.004; red vs. yellow, ***X***^**2**^ = 2.425, *p* = 0.119). No difference in occupational distribution was observed in the three groups. All psychological indicators related to depression were significantly different in the three groups after correction of age and sex as covariates (Table 1). The PROVE scores for depressive symptoms, and mentalization failure were significantly different in all the three groups (PROVE-DS, F_1,2_ = 88.955, *p* < 0.001; PROVE-MC, F_1,2_ = 18.055, *p* < 0.001). Furthermore, there was a significant suicide risk (PROVE-SR, F_1,2_ = 15.536, *p* < 0.001) difference between the red and the other two groups (red vs. green, *p* < 0.001; red vs. yellow, *p* < 0.001), while no difference was observed between the yellow and green groups (*p* = 0.509). The PROVE scores of ACE and resilience (PROVE-ACE, F_1,2_ = 9.451, *p* < 0.001; PROVE-KRQ, F_1,2_ = 10.89, *p* < 0.001) differed between the green and the other two groups (PROVE-ACE, green vs. yellow, *p* = 0.011; green vs. red, *p* < 0.001; PROVE-KRQ, green vs. yellow, *p* = 0.001; green vs. red, *p* < 0.001), and the ACE and resilience scores between yellow and red groups were not significantly different (PROVE-ACE, *p* = 0.274; PROVE-KRQ, *p* = 1; Figure 3C). The red group showed the highest depressive symptoms, suicide risk, and mentalization failure. There were differences in the attachment types of the participants of these groups (*X*^2^ = 29.435, *p* < 0.001); the proportion of disorganized type was high in the red group (Figure 3F).

**Table 1:** Demographic and psycho-social characteristics of groups by PROtective and Vulnerable factors battEry questionnaire.

	**Green (*n* = 20)**	**Yellow** **(*n* = 30)**	**Red (*n* = 23)**	***F/X^**2**^***	***p*-value**
Age, mean (SD)	38.7 (10.26)	32.9 (6.26)	30.9 (8.11)	5.357	0.007^**^
Sex				10.51	0.005^**^
Male, *n* (%)	8 (40%)	17 (56.7%)	3 (13%)		
Female, *n* (%)	12 (60%)	13 (43.3%)	20 (86.7%)		
Occupation				3.325	0.505
Presence, *n* (%)	13 (65%)	24 (80%)	14 (60.9%)		
None, *n* (%)	3 (15%)	4 (13.3%)	4 (17.4%)		
Student, *n* (%)	4 (20%)	2 (6.7%)	5 (21.7%)		
Psychiatric disease history				8.574	0.014^*^
Presence, *n* (%)	1 (5%)	11 (23.3%)	10 (43.5%)		
None, *n* (%)	19 (95%)	24 (76.7%)	13 (56.5%)		
PROVE-DS	4.4 (2.98)	21.7 (7.01)	31.57 (7.39)	88.955	<0.001^***^
PROVE-SR	0.7 (1.34)	2.67 (1.9)	6.8 (5.18)	15.536	<0.001^***^
PROVE-KRQ	203.1 (23.15)	174.3 (21.77)	166.22 (25.73)	10.89	<0.001^***^
PROVE-ACE	12.25 (9.22)	30.6 (17.34)	40.52 (22.04)	9.451	<0.001^***^
PROVE-MC	21 (6.95)	26.5 (6.55)	32.48 (7.22)	18.055	<0.001
PROVE-AAT-anxiety	19.35 (8.57)	33.53 (8.93)	42.35 (12.76)	19.174	<0.001
PROVE-AAT-avoidance	30.4 (8.98)	32.43 (7.9)	40.61 (7.99)	6.467	0.003
Attachment type				29.435	<0.001
Stable	16	13	3		
Anxiety	0	10	5		
Avodance	3	1	3		
Disorganized	1	6	12		

*Analysis of variance was performed on the differences in age by group, and chi-square analysis was performed for group-specific differences in other categorical variables. Analysis of covariance was done for the differences in PROVE sub-scales with covariates of age and sex. SD, standard deviation. PROtective and Vulnerable factors battEry questionnaire; DS, depressive symptomatology; SR, suicide risk; KRQ, Korean resilience quotient; ACE, adverse childhood experience, MC, mentalization capacity problem; AAT, adult attachment type*.

* *p < 0.05*,

** *p < 0.01*,

*** *p < 0.001*.

### Factors Influencing Resilience in Whole Participants

A univariate linear regression analysis was conducted to understand the effects that enhance resilience, using resilience total score (PROVE-KRQ) as the dependent variable (Table 2). Independent variables were age, sex, childhood trauma (PROVE-ACE score), mentalization (PROVE-MC score), attachment (disorganized type), and AUCg. All factors but sex were identified to influence on the resilience according to univariate regression analysis (age, β = 0.266, *p* = 0.023; adverse childhood experience, β = −0.422, *p* < 0.001; mentalization failure, β = −0.425, *p* < 0.001; disorganized attachment, β = −0.518, *p* < 0.001; AUCg, β = 0.31, *p* = 0.008). In the multivariate regression analysis, disorganized attachment (β = −0.359, *p* = 0.002) was verified as valid resilience predictors. The explain variance of the model was 42.9%.

**Table 2:** Linear regression analysis of resilience score in whole participants.

	**Univariate analysis**		**Multivariate analysis**	
	**Standardized Beta**	***p*-value**	**Standardized Beta**	***p*-value**
PROVE-ACE	−0.422	<0.001^***^	−0.22	0.059
PROVE-MC	−0.425	<0.001^***^	−0.207	0.055
Attachment type (disorganized)	−0.518	<0.001^***^	−0.359	0.002^**^
AUCg	0.31	0.008^**^	0.073	0.504
Age	0.266	0.023^*^	0.053	0.611
Sex (male)	0.891	0.348	0.003	0.979

*PROVE, PROtective and Vulnerable factors battEry questionnaire; ACE, adverse childhood experience; MC, mentalization capacity problem; AUCg, area under curve with respect to the ground of post-awakening cortisol concentrations*.

* *p < 0.05*,

** *p < 0.01*,

*** *p < 0.001*.

### Factors Influencing Resilience in a Risk Group and Healthy Group

The univariate and multivariate linear regression analyses were conducted in the red and green groups to identify the factors that affect resilience in risky or healthy mental health groups respectively (Table 3; Supplementary Table 1). In the risky group, the disorganized attachment (β = −0.91, *p* < 0.001) negatively influenced resilience in the multivariate regression analysis. This model accounted for a 73.2% variance for this outcome. In the healthy group, all factors that we considered did not have a significant effect on resilience.

**Table 3:** Linear regression analysis of resilience score in the red group.

	**Univariate analysis**		**Multivariate analysis**	
***N* = 23**	**Standardized Beta**	***p*-value**	**Standardized Beta**	***p*-value**
PROVE-ACE	−0.354	0.102	0.041	0.792
PROVE-MC	−0.297	0.22	−0.02	0.897
Attachment type (disorganized)	−0.845	<0.001^***^	−0.91	<0.001^***^
AUCg	0.319	0.145	−0.63	0.538
Age	−0.234	0.283	−0.471	0.004^**^
Sex (male)	−0.121	0.581	−0.238	0.13

*PROVE, PROtective and Vulnerable factors battEry questionnaire; ACE, adverse childhood experience; MC, mentalization capacity problem; AUCg, area under the curve with respect to the ground of post-awakening cortisol concentrations*.

** *p < 0.01*,

*** *p < 0.001*.

### Factors Influencing Resilience and Depressive Symptoms in Groups With ACEs

The univariate and multivariate linear regression analyses were conducted on the participants with ACEs to identify the factors that affect resilience and depressive symptoms each (Tables 4, 5). In terms of the resilience, the mentalization failure and disorganized attachment were indicated as possible negative influencers in univariate regression analysis (mentalization failure, β = −0.394, *p* = 0.045; disorganized attachment, β = −0.493, *p* = 0.012), although no factors were identified in the multivariate regression analysis (Table 4). These two factors also has been found to affect participants who have ACEs toward increasing depressive symptoms (mentalization failure, β = 0.533, *p* = 0.004; disorganized attachment, β = 0.463, *p* = 0.016) in univariate regression analysis (Table 5). Especially, the multivariate regression analysis verified that mentalization failure can be a possible influencing factor to increase depressive symptoms in people with ACEs (β = 0.41, *p* = 0.038). The explained variance of the model of resilience and depressive symptoms through multivariate regression analysis were 30.5 and 39.4%, respectively.

**Table 4:** Linear regression analysis of resilience score in the subjects with ACE.

	**Univariate analysis**		**Multivariate analysis**	
***N* = 29**	**Standardized Beta**	***p*-value**	**Standardized Beta**	***p*-value**
PROVE-MC	−0.394	0.045^*^	−0.221	0.28
Attachment type (disorganized)	−0.493	0.012^*^	−0.337	0.123
AUCg	0.312	0.134	0.195	0.319
Age	−0102	0.6	−0.067	0.73
Sex (male)	0.018	0.928	−0.123	0.55

*ACE, adverse childhood experience; PROVE, PROtective and Vulnerable factors battEry questionnaire; MC, mentalization capacity problem; AUCg, area under the curve with respect to the ground of post-awakening cortisol concentrations*.

* *p < 0.05*.

**Table 5:** Linear regression analysis of depression score in subjects with ACE.

	**Univariate analysis**		**Multivariate analysis**	
***N* = 29**	**Standardized Beta**	***p*-value**	**Standardized Beta**	***p*-value**
PROVE-MC	0.533	0.004^**^	0.41	0.038^*^
Attachment type (disorganized)	0.463	0.016^*^	0.292	0.152
AUCg	−0.038	0.856	0.087	0.63
Age	−0.016	0.933	0.003	0.987
Sex (male)	−0.234	0.222	−0.17	0.379

*ACE, adverse childhood experience; PROVE, PROtective and Vulnerable factors battEry questionnaire; MC, mentalization capacity problem; AUCg, area under the curve with respect to the ground of post-awakening cortisol concentrations*.

* *p < 0.05*,

** *p < 0.01*.

### Stress Hormones and Psycho-Social Factors

**Figure 4:**
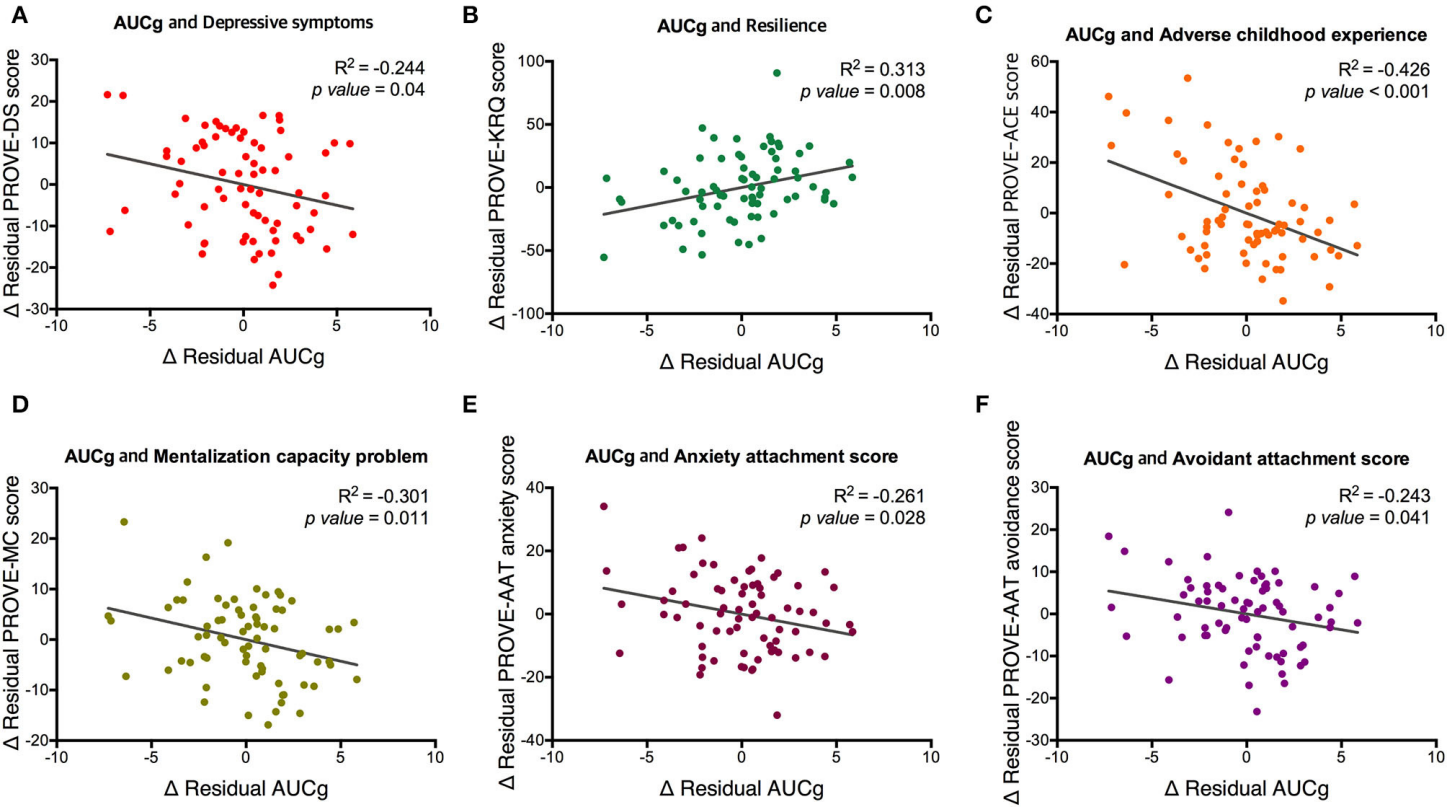
Partial correlation between AUCg and psychological factors related to depression with covariates of age and sex. Associations of AUCg and **(A)** depressive symptoms, **(B)** resilience, **(C)** adverse childhood experience, **(D)** mentalization capacity problem, **(E)** anxiety attachment score and **(F)** avoidance attachment score are represented. PROVE, PROtective and Vulnerable factors battEry questionnaire; KRQ, Korean Resilience Quotient; ACE, adverse childhood experience MC, mentalization capacity problem; AAT, adult attachment type; AUCg, area under the curve with respect to the ground of post-awakening cortisol concentrations.

The AUCg was different by PROVE group, and there was a significant difference between green and red groups (F_1,2_ = 3.715, *p* = 0.029; green vs. red (*post-hoc*), *p* = 0.025; Figure 3A). In addition, based on the presence or absence of ACE, AUCg showed significant differences (F_**1,1**_ = 5.5, *p* = 0.022; Figure 3C). AUCi did not differ according to the PROVE group or presence or absence of ACE (PROVE group, F_1,2_ = 2.568, *p* =0.084; ACE group, F_1,1_ = 1.708, *p* = 0.196; Figures 3B,D). Both AUCg and AUCi differed depending on the resilience group (AUCg, F_1,2_ = 3.44, *p* = 0.038; AUCi, F_1,2_ = 4.248, *p* = 0.018; Figures 3E,F). The high resilience group showed increased AUCi as compared to moderate or low resilience groups (high vs. moderate, *p* = 0.025; high vs. low, *p* = 0.018; Figure 3F). The difference of AUCg between each group was not observed after Bonferroni correction (high vs. moderate, *p* = 1 high vs. low, *p* = 0.144; moderave vs. low, *p* = 0.093). Sl did not observe group-specific differences in both the PROVE group, ACE group, resilience group analysis (Supplementary Table 2). AUCg was correlated with a number of depression-related vulnerability-protecting factors after adjustment of age and sex as covariates (Figure 4). The resilience score was positively correlated with AUCg (*R*^2^ = 0.313, *p* = 0.008; Figure 4B), but the scores for depressive symptoms (*R*^2^ = −0.244, *p* = 0.04; Figure 4A), ACE (*R*^2^ = −0.426, *p* < 0.001; Figure 4C), mentalization (*R*^2^ = −0.301, *p* = 0.011; Figure 4D), attachment anxiety (*R*^2^ = −0.61, *p* = 0.028; Figure 4E) and attachment avoidance (*R*^2^ = −0.243 *p* = 0.041; Figure 4E) showed negative correlation.

## Discussion

This study aimed to identify the factors contributing to resilience in the entire group, each group, and the ACE group from a bio-psycho-social perspective. We also tried to analyze protective factors against depression in the ACE group. In addition, we investigated whether AUCg could be a promising biomarker related to depression in context with resilience. This study suggests that disorganized attachment has a major influence on resilience as we validated it at the whole participants level as well as in risky groups or ACE. Mentalization problem also negatively affects resilience in the whole PROVE group and people with ACEs. In addition, mentalization problem is associated with increased depressive symptoms in people with ACEs. Furthermore, we found that the post-awakening cortisol concentration (AUCg) is decreased in the risky group and the ACE group, and CAR (AUCi) is increased in the high resilience group. The AUCg was positively correlated with high resilience and negatively correlated with depressive symptoms and high vulnerable factor scores.

The relationship between attachment, mentalization, and resilience has been described in several studies (21, 26, 55). The concept of resilience has basically consisted of adversity and positive adaptation, and both attachment and mentalization can be prerequisites for positive adaptation (19). According to attachment theory, attachment representation results in inner working models into adulthood, and our behaviors in social and stressful situations are considered a manifestation of it (56). Securely attached people will expect a secure base to which they can return for safety and comfort when distressed while disorganized attached people lack any attachment strategy (56). Meta-analysis showed that secure attachment is significantly associated with resilience and it might be the core factor in positive adaptation (19). Mentalization capacity is also a type of higher-order cognition that enables positive adaptation after adversity. Mentalization is basically generated through mirroring and communication with the caregiver with a secure attachment in childhood. However, people with early life stress or insecure attachment can improve their mentalization capacity through evidence-based psychosocial interventions. This study suggests that the mentalization capacity in the ACE group shows a considerable effect on depressive symptoms. Therefore, treatments that promote mentalization ultimately help cultivate resilience and prevent depression or trigger recovery. This strategy has been originally developed to treat patients with a borderline personality disorder but has also been effective for depression, especially for individuals with ACEs and vulnerable factors. In addition, when an adult with insecure attachment raises a child, improved mentalization capacity is needed to establish a good attachment with the child. In this respect, mentalization-based intervention, such as mentalization-based treatment (MBT), can be used as an important method to enhance acquired resilience. MBT is a manualized treatment developed by Anthony Bateman and Peter Fonagy to increase the mentalizing ability of patients (57). In MBT, the mentalizing capacity is enhanced through emphatic validation within the therapeutic relaxation, and through techniques that directly focus on mentalizing (58). Edel et al. (59) and Bo et al. (60), reported improved mentalization after a 6-week and 1-year treatment, respectively, which proved with improved mentalization task and reflective functioning questionnaire for youth scores. Another study showed that MBT adherence and competence predicted higher mentalizing scores (61). Taken together, MBT has been demonstrated to be effective in increasing mentalizing capacity, which verified its usefulness as a method to enhance acquired resilience.

In terms of biological factors, AUCg showed a significant influence on resilience in the univariate regression analysis, but not in multivariate regression analysis. In the risky group and ACE participants, AUCg was blunted as compared to the healthy group and those without ACE, respectively. AUCi was also blunted in the participants with moderate or low resilience as compared to those with high resilience. Further, AUCg was significantly correlated with protective-vulnerable factors of depression, such as resilience, mentalization, and attachment. It showed a positive correlation with resilience score and negatively correlated with depressive symptoms ACE, mentalization failure, and anxiety and avoidance attachment scores. Taken together, this study shows that post-awakening salivary cortisol concentration and response tended to be blunted when resilience was low and other vulnerability factors or depressive symptoms were high.

Considerable heterogeneity was found in a previous study about linking psychosocial functioning to post-awakening salivary cortisol indices (62). According to the meta-analysis, higher AUCg was associated with depression, and lower AUCg was associated with posttraumatic stress (63). In the case of AUCi, a higher value was associated with general life stress or posttraumatic stress, and a lower value was associated with fatigue or burnout. Overall, AUCg had a greater effect size than AUCi, which implicates that the total cortisol output (AUCg) of the post-awakening period may be significantly affected as the psychosocial predictor than the dynamic increase (AUCi). It suggests that chronic worse psychosocial functioning may blunt the overall output of the CAR system while the dynamic component remained intact (63). This result is consistent with the result of the current study that the lower AUCg is associated with a higher risk of psychosocial factors.

In the previous study that investigated suicide risk and resilience, lower AUCg was related to less resilience and indirectly affected suicide risk (64). Likewise, it has been reported that childhood trauma is associated with lower AUCg (65). Here, repetitive stressful experiences can increase the allostatic load, causing HPA axis dysregulation, which in turn may lead to decreased resilience capacity. There are also reports that suggest that high resilience is associated with high AUCi in healthy individuals (34); such a relationship was also determined in this study (Figure 4). Further studies are needed to evaluate whether an ACE individual with low resilience recovers CAR by enhancing resilience through psychosocial interventions, such as MBT. To investigate CAR as a protective biomarker for patients with depressive disorder, further studies are required.

## Limitation

A major limitation of this study is that health controls and disease groups were not separately gathered, and the age and sex of subjects have not been matched. There can be confounding factors as a randomized controlled recruitment procedure was not incorporated in this retrospective study; there were significant differences in participants' age, sex, and psychiatric disorder history between different PROVE groups. Even within the actual population, it is likely that women or younger people are likely to belong to the risky group, but it will be difficult to conclude with this research, including uncontrolled small samples. Although age and sex differences were corrected as covariates, there would not be an analysis to explore the exact difference between groups as randomized controlled. A follow-up prospective study is needed with the randomized controlled recruitment process for clinical and control groups.

The other major limitation of this study is uncertainty in the accuracy of saliva samples collection time and the lack of other covariates for interpretation of salivary cortisol. Although all participants were informed of the importance of precise collection time, and S1 showed no differences by group, objective data on saliva collection time was not obtained. In addition to the time of awakening, other information necessary for cortisol analysis, such as sleep duration, quality, light level, season, priority day experience, socioeconomic status, habitual saving history, heavy drinking history, and body-mass-index. were also not collected. Although the data that were determined to show a complete delayed pattern were excluded, the results related to salivary cortisol in this study should be read with a great limit to the interpretation because it could not include important covariates in the analysis. In subsequent prospective studies, we will definitely acquire this information together and include it in the analysis and interpretation.

In addition, the Cronbach's alpha value of PROVE-MC, which measures mentalization, is 0.68, which is lower than 0.7. Therefore, the reliability is thought to be relatively low. We will revise the scale in the future to increase its internal consistency.

The last one is that the relationship of resilience was investigated only with cortisol response as a biomarker. Although there are several resilience-related biomarkers, such as heart-rate variability, neuroinflammation, epigenetic changes, and genetic polymorphisms, they are not used in clinical practice due to a lack of integrated information (22, 66–69). In future studies, it is necessary to evaluate resilience with more multi-faceted relevant biomarkers.

## Conclusion

In this study, the relationship of resilience with ACE, attachment, mentalization capacity, and salivary cortisol response was comprehensively explored in participants with various levels of depression risk. Resilience is negatively affected by disorganized attachment and mentalization failure, and improving these factors can lead to the enhancement of resilience. In addition, the advance in mentalization may ameliorate depression in people with ACEs. With respect to biological factors, post-awakening cortisol concentration blunted AUCg is associated with low resilience, high depressive symptoms, and high vulnerability factor scores. It is worth verifying the possibility of biological markers through a more precisely designed study.

## Data Availability Statement

The raw data supporting the conclusions of this article will be made available by the authors, without undue reservation.

## Ethics Statement

The studies involving human participants were reviewed and approved by Institutional Review Board of Gangnam Severance Hospital, Yonsei University College of Medicine (Approval No. 3-2021-0451). The patients/participants provided their written informed consent to participate in this study.

## Author Contributions

J-HS and SJ devised the project, the main ideas, and proof of the outline. S-WC and J-YL examined participants and acquired and organized the data. RA conducted salivary cortisol analyses. SJ, S-WC, and RA drafted the manuscript. SJ performed the statistical analysis and prepared figures. JK contributed to interpreting the results and worked on the manuscript. J-HS supervised the project. All authors discussed the results, commented on the manuscript, contributed to the article, and approved the submitted version.

## Funding

This work was supported by the Korea Medical Device Development Fund of the Korean government (the Ministry of Science and ICT, the Ministry of Trade, Industry and Energy, the Ministry of Health & Welfare, the Ministry of Food and Drug Safety) (Project Number: 9991006856, KMDF_PR_20200901_0186).

## Conflict of Interest

J-HS is a professor of Yonsei University and the CEO of Minds.AI which has been established since November 2019 as a research and development company for mental health in Korea. SJ, S-WC, RA, and J-YL are employed by Minds.AI, Co. Ltd. The remaining author declares that the research was conducted in the absence of any commercial or financial relationships that could be construed as a potential conflict of interest.

## Publisher's Note

All claims expressed in this article are solely those of the authors and do not necessarily represent those of their affiliated organizations, or those of the publisher, the editors and the reviewers. Any product that may be evaluated in this article, or claim that may be made by its manufacturer, is not guaranteed or endorsed by the publisher.

## References

[1] Torres-Berrío A Issler O Parise EM Nestler EJ. Unraveling the epigenetic landscape of depression: focus on early life stress. Dialogues Clin Neurosci. (2019) 21:341–57. 10.31887/DCNS.2019.21.4/enestler 31949402 PMC6952747

[2] LeMoult J Humphreys KL Tracy A Hoffmeister JA Ip E Gotlib IH. Meta-analysis: exposure to early life stress and risk for depression in childhood and adolescence. J Am Acad Child Adolesc Psychiatry. (2020) 59:842–55. 10.1016/j.jaac.2019.10.011 31676392 PMC11826385

[3] Nelson J Klumparendt A Doebler P Ehring T. Childhood maltreatment and characteristics of adult depression: meta-analysis. Br J Psychiatry. (2017) 210:96–104. 10.1192/bjp.bp.115.180752 27908895

[4] Carr CP Martins CM Stingel AM Lemgruber VB Juruena MF. The role of early life stress in adult psychiatric disorders: a systematic review according to childhood trauma subtypes. J Nerv Ment Dis. (2013) 201:1007–20. 10.1097/NMD.0000000000000049 24284634

[5] Mueller I Tronick E. early life exposure to violence: developmental consequences on brain and behavior. Front Behav Neurosci. (2019) 13:156. 10.3389/fnbeh.2019.00156 31338031 PMC6629780

[6] du Plessis MR Smeekens S Cillessen AHN Whittle S Güroglu B. Bullying the brain? longitudinal links between childhood peer victimization, cortisol, and adolescent brain structure. Front Psychol. (2019) 9:2706. 10.3389/fpsyg.2018.02706 30692951 PMC6340095

[7] Wilson KR Hansen DJ Li MJA. The traumatic stress response in child maltreatment and resultant neuropsychological effects. Aggress Violent Behav. (2011) 16:87–97. 10.1016/j.avb.2010.12.007

[8] Twardosz S Lutzker JRJA. behavior v. Child maltreatment and the developing brain: A review of neuroscience perspectives. Aggress Violent Behav. (2010) 15:59–68. 10.1016/j.avb.2009.08.003

[9] Iob E Baldwin JR Plomin R Steptoe A. Adverse childhood experiences, daytime salivary cortisol, and depressive symptoms in early adulthood: a longitudinal genetically informed twin study. Transl Psychiatry. (2021) 11:420. 10.1038/s41398-021-01538-w 34354040 PMC8342545

[10] Koss KJ Gunnar MR. Annual research review: early adversity, the hypothalamic-pituitary-adrenocortical axis, and child psychopathology. J Child Psychol Psychiatry. (2018) 59:327–46. 10.1111/jcpp.12784 28714126 PMC5771995

[11] Sapolsky RM Romero LM Munck AU. How do glucocorticoids influence stress responses? integrating permissive, suppressive, stimulatory, and preparative actions. Endocr Rev. (2000) 21:55–89. 10.1210/edrv.21.1.0389 10696570

[12] Stetler C Miller GE. Depression and hypothalamic-pituitary-adrenal activation: a quantitative summary of four decades of research. Psychosom Med. (2011) 73:114–26. 10.1097/PSY.0b013e31820ad12b 21257974

[13] David M Fergusson PEM. Childhood Sexual Abuse: An Evidenced Based Perspective. Thousand Oaks, CA: SAGE Publications, Inc (1999). 10.4135/9781452205540

[14] Stevenson J Psychiatry, Disciplines A. The treatment of the long-term sequelae of child abuse. J Child Psychol Psychiatry. (1999) 40:89–111. 10.1111/1469-7610.00425 10102727

[15] McGloin JM Widom CSJD. Resilience among abused and neglected children grown up. Dev Psychopathol. (2001) 13:1021–38. 10.1017/S095457940100414X 11771905

[16] Iacoviello BM Charney DS. Cognitive and Behavioral Components of Resilience to Stress. Stress resilience: Elsevier (2020). p. 23–31. 10.1016/B978-0-12-813983-7.00002-1

[17] Babić R Babić M Rastović P Curlin M Šimic J Mandic K. Resilience in Health and Illness. Psychiatr Danub. (2020) 32:226–3. Available online at: https://scholar.google.com/scholar_lookup?title=Resilience+in+Health+and+Illness&author=Babi%C4%87,+R.&author=Babi%C4%87,+M.&author=Rastovi%C4%87,+P.&author=%C4%86urlin,+M.&author=%C5%A0imi%C4%87,+J.&author=Mandi%C4%87,+K.&author=Pavlovi%C4%87,+K.&publication_year=2020&journal=Psychiatr.+Danub.&volume=32&pages=226%E2%80%93232. 32970640

[18] Leung DY Chan AC Ho GWK. Resilience of emerging adults after adverse childhood experiences: a qualitative systematic review. Trauma Violence Abuse. (2022) 23:163–81. 10.1177/1524838020933865 32588765

[19] Darling Rasmussen P Storebø OJ Løkkeholt T Voss LG Shmueli-Goetz Y Bojesen AB. Attachment as a core feature of resilience: a systematic review and meta-analysis. Psychol Rep. (2019) 122:1259–96. 10.1177/0033294118785577 29958512

[20] Lau WK Tai AP Chan JN Lau BW Geng XJP. Integrative psycho-biophysiological markers in predicting psychological resilience. Psychoneuroendocrinology. (2021) 129:105267. 10.1016/j.psyneuen.2021.105267 34015682

[21] Jackson R Watkin CJ. The resilience inventory: seven essential skills for overcoming life's obstacles and determining happiness. Select Dev Rev. (2004) 20:13–7. Available online at: https://scholar.google.com/scholar_lookup?hl=en&volume=20&publication_year=2004&pages=13-17&issue=6&author=R.+Jackson&author=C.+Watkin&title=The+resilience+inventory%3A+Seven+essential+skills+for+overcoming+life%27s+obstacles+and+determining+happiness

[22] Kalisch R Müller MB Tüscher O. A conceptual framework for the neurobiological study of resilience. Behav Brain Sci. (2015) 38:e92. 10.1017/S0140525X15000023 25158686

[23] Bateman AW Fonagy PE. Handbook of Mentalizing in Mental Health Practice. Washington, DC: American Psychiatric Publishing, Inc. (2012).

[24] Kalisch R Baker DG Basten U Boks MP Bonanno GA Brummelman E. The resilience framework as a strategy to combat stress-related disorders. Nat Hum Behav. (2017) 1:784–90. 10.1038/s41562-017-0200-8 31024125

[25] Allen JG Fonagy P. The Handbook of Mentalization-Based Treatment. Chichester: John Wiley and Sons (2006). 10.1002/9780470712986

[26] Fonagy P Campbell C. Mentalizing, attachment and epistemic trust: how psychotherapy can promote resilience. Psychiatr Hung. (2017) 32:283-7. Available online at: https://scholar.google.com/scholar?hl=en&as_sdt=0%2C5&q=Mentalizing%2C+attachment+and+epistemic+trust%3A+how+Q22+psychotherapy+can+promote+resilience.&btnG= 29135441

[27] Bouchard M-A Target M Lecours S Fonagy P Tremblay L-M Schachter A. Mentalization in adult attachment narratives: reflective functioning, mental states, and affect elaboration compared. Psychoanalytic Psychol. (2008) 25:47–66. 10.1037/0736-9735.25.1.47

[28] Fonagy P Luyten P. Attachment, Mentalizing, and the Self (2018).

[29] Fonagy P Bateman AW. Adversity, attachment, and mentalizing. Compr Psychiatry. (2016) 64:59–66. 10.1016/j.comppsych.2015.11.006 26654293

[30] Roche DN Runtz MG Hunter MAJJoIV. Adult attachment: a mediator between child sexual abuse and later psychological adjustment. J Interpers Violence. (1999) 14:184–207. 10.1177/088626099014002006

[31] Shapiro DL Levendosky AA. Adolescent survivors of childhood sexual abuse: the mediating role of attachment style and coping in psychological and interpersonal functioning. Child Abuse Negl. (1999) 23:1175–91. 10.1016/S0145-2134(99)00085-X 10604070

[32] Deighton S Neville A Pusch D Dobson K. Biomarkers of adverse childhood experiences: a scoping review. Psychiatry Res. (2018) 269:719–32. 10.1016/j.psychres.2018.08.097 30273897

[33] Fogelman N Canli T. Early life stress and cortisol: a meta-analysis. Horm Behav. (2018) 98:63–76. 10.1016/j.yhbeh.2017.12.014 29289660

[34] Lai JCL Leung MOY Lee DYH Lam YW Berning K. Biomarking trait resilience with salivary cortisol in chinese undergraduates. Front Psychol. (2020) 11:536510. 10.3389/fpsyg.2020.536510 33192778 PMC7649282

[35] García-León M Pérez-Mármol JM Gonzalez-Pérez R García-Ríos MDC Peralta-Ramírez MI. Relationship between resilience and stress: perceived stress, stressful life events, HPA axis response during a stressful task and hair cortisol. Physiol Behav. (2019) 202:87–93. 10.1016/j.physbeh.2019.02.001 30726720

[36] Southwick SM Vythilingam M Charney DS. The psychobiology of depression and resilience to stress: implications for prevention and treatment. Annu Rev Clin Psychol. (2005) 1:255–91. 10.1146/annurev.clinpsy.1.102803.143948 17716089

[37] Gilbert PJS. Depression and stress: a biopsychosocial exploration of evolved functions and mechanisms. Stress. (2001) 4:121–35. 10.3109/10253890109115726 22432132

[38] Schotte CK Van Den Bossche B De Doncker D Claes S Cosyns P. A biopsychosocial model as a guide for psychoeducation and treatment of depression. Depress Anxiety. (2006) 23:312–24. 10.1002/da.20177 16688730

[39] Laurent M Bustany P de Tychey C Lighezzolo-Alnot J. Psychobiological resilience: a longitudinal qualitative exploratory approach. J Trauma Stress Disorder Treat. (2017) 4:2. 10.4172/2324-8947.1000180

[40] Li ET Carracher E Bird T. Linking childhood emotional abuse and adult depressive symptoms: the role of mentalizing incapacity. Child Abuse Negl. (2020) 99:104253. 10.1016/j.chiabu.2019.104253 31812024

[41] Dagan O Facompré CR Bernard K. Adult attachment representations and depressive symptoms: a meta-analysis. J Affect Disord. (2018) 236:274–90. 10.1016/j.jad.2018.04.091 29751243

[42] Lee J-Y Choi S-W Jang S-A Ryu J-S Shin H-K Sim J-Y. Development of the battery test for screening of depression and mental health: protective and vulnerable factors battery test (PROVE). J Korean Neuropsychiatr Assoc. (2021) 60:143–57. 10.4306/jknpa.2021.60.2.143

[43] Kim J Seok JH Choi K Jon DI Hong HJ Hong N. The protective role of resilience in attenuating emotional distress and aggression associated with early-life stress in young enlisted military service candidates. J Korean Med Sci. (2015) 30:1667–74. 10.3346/jkms.2015.30.11.1667 26539013 PMC4630485

[44] JH K. Resilience - A Pleasant Secret Replacing Hardship With Fortune. Goyang: Wisdomhouse (2011).

[45] Loh JM Schutte NS Thorsteinsson EB. Be happy: the role of resilience between characteristic affect and symptoms of depression. J Happiness Stud. (2014) 15:1125–38. 10.1007/s10902-013-9467-2

[46] Chapman DP Whitfield CL Felitti VJ Dube SR Edwards VJ. Anda RF. Adverse childhood experiences and the risk of depressive disorders in adulthood. J Affect Disord. (2004) 82:217–25. 10.1016/j.jad.2003.12.013 15488250

[47] Clow A Hucklebridge F Stalder T Evans P Thorn L. The cortisol awakening response: more than a measure of HPA axis function. Neurosci Biobehav Rev. (2010) 35:97–103. 10.1016/j.neubiorev.2009.12.011 20026350

[48] Pruessner JC Wolf OT Hellhammer DH Buske-Kirschbaum A von Auer K Jobst S. Free cortisol levels after awakening: a reliable biological marker for the assessment of adrenocortical activity. Life Sci. (1997) 61:2539–49. 10.1016/S0024-3205(97)01008-4 9416776

[49] Stadler T Kirschbaum C Kudielka B Adam E Wüst S Dockray S. Assessment of the cortisol awakening response: expert consensus guidelines. Psychoneuroendocrinology. (2015) 63:414. 10.1016/j.psyneuen.2015.10.010 26563991

[50] Wüst S Wolf J Hellhammer DH Federenko I Schommer N Kirschbaum C. The cortisol awakening response - normal values and confounds. Noise Health. (2000) 2:79–88. 12689474

[51] Okun ML Krafty RT Buysse DJ Monk TH Reynolds CF Begley A. What constitutes too long of a delay? determining the cortisol awakening response (CAR) using self-report and PSG-assessed wake time. Psychoneuroendocrinology. (2010) 35:460–8. 10.1016/j.psyneuen.2009.08.017 19762158 PMC2823961

[52] Ahn RS Lee YJ Choi JY Kwon HB Chun SI. Salivary cortisol and DHEA levels in the Korean population: age-related differences, diurnal rhythm, and correlations with serum levels. Yonsei Med J. (2007) 48:379–88. 10.3349/ymj.2007.48.3.379 17594144 PMC2628086

[53] Kim MS Lee YJ Ahn RS. Day-to-day differences in cortisol levels and molar cortisol-to-DHEA ratios among working individuals. Yonsei Med J. (2010) 51:212–8. 10.3349/ymj.2010.51.2.212 20191012 PMC2824866

[54] Kobayashi H Miyazaki Y. Distribution characteristics of salivary cortisol measurements in a healthy young male population. J Physiol Anthropol. (2015) 34:30. 10.1186/s40101-015-0068-0 26286592 PMC4543482

[55] Bak PL Midgley N Zhu JL Wistoft K Obel C. The Resilience Program: preliminary evaluation of a mentalization-based education program. Front Psychol. (2015) 6:753. 10.3389/fpsyg.2015.00753 26136695 PMC4468359

[56] Ainsworth MDS Blehar MC Waters EJH Lawlence Erlbaum Associates Wall S. Patterns of Attachment: A Psychological Study of the Strange Situation. Hillsdale, NJ: Lawrence Erlbaum (1978).

[57] Bateman A Fonagy P. Mentalization Based Treatment for Personality Disorders: A Practical Guide. Oxford, UK: Oxford University Press (2016). 10.1093/med:psych/9780199680375.001.0001

[58] Volkert J Hauschild S Taubner S. Mentalization-based treatment for personality disorders: efficacy, effectiveness, and new developments. Curr Psychiatry Rep. (2019) 21:25. 10.1007/s11920-019-1012-5 30852694

[59] Edel MA Raaff V Dimaggio G Buchheim A Brüne M. Exploring the effectiveness of combined mentalization-based group therapy and dialectical behaviour therapy for inpatients with borderline personality disorder–a pilot study. Br J Clin Psychol. (2017) 56:1–15. 10.1111/bjc.12123 27897326

[60] Bo S Sharp C Beck E Pedersen J Gondan M Simonsen E. First empirical evaluation of outcomes for mentalization-based group therapy for adolescents with BPD. Personal Disord. (2017) 8:396–401. 10.1037/per0000210 27845526

[61] Möller C Karlgren L Sandell A Falkenström F Philips B. Mentalization-based therapy adherence and competence stimulates in-session mentalization in psychotherapy for borderline personality disorder with co-morbid substance dependence. Psychother Res. (2017) 27:749–65. 10.1080/10503307.2016.1158433 27093128

[62] Chida Y. Steptoe A. Cortisol awakening response and psychosocial factors: a systematic review and meta-analysis. Biol Psychol. (2009) 80:265–78. 10.1016/j.biopsycho.2008.10.004 19022335

[63] Boggero IA Hostinar CE Haak EA Murphy ML Segerstrom SC. Psychosocial functioning and the cortisol awakening response: meta-analysis, P-curve analysis, and evaluation of the evidential value in existing studies. Biol Psychol. (2017) 129:207–30. 10.1016/j.biopsycho.2017.08.058 28870447 PMC5673546

[64] O'Connor DB Branley-Bell D Green JA Ferguson E O'Carroll RE O'Connor RC. Resilience and vulnerability factors influence the cortisol awakening response in individuals vulnerable to suicide. J Psychiatr Res. (2021) 142:312–20. 10.1016/j.jpsychires.2021.08.006 34419751

[65] O'Connor DB Branley-Bell D Green JA Ferguson E O'Carroll RE O'Connor RC. Effects of childhood trauma, daily stress, and emotions on daily cortisol levels in individuals vulnerable to suicide. J Abnorm Psychol. (2020) 129:92–107. 10.1037/abn0000482 31657598

[66] An E Nolty AAT Amano SS Rizzo AA Buckwalter JG Rensberger J. Heart rate variability as an index of resilience. Mil Med. (2020) 185:363–9. 10.1093/milmed/usz325 31642481

[67] Carnevali L Koenig J Sgoifo A Ottaviani C. Autonomic and brain morphological predictors of stress resilience. Front Neurosci. (2018) 12:228. 10.3389/fnins.2018.00228 29681793 PMC5897537

[68] Dudek KA Kaufmann FN Lavoie O Menard C. Central and peripheral stress-induced epigenetic mechanisms of resilience. Curr Opin Psychiatry. (2021) 34:1–9. 10.1097/YCO.0000000000000664 33141775

[69] Wu Z Xiao L Wang H Wang G. Neurogenic hypothesis of positive psychology in stress-induced depression: Adult hippocampal neurogenesis, neuroinflammation, and stress resilience. Int Immunopharmacol. (2021) 97:107653. 10.1016/j.intimp.2021.107653 33915495

