# Supplementary material for: Relationship of Resilience Factors With Biopsychosocial Markers Using a Comprehensive Home Evaluation Kit for Depression and Suicide Risk: A Real-World Data Analysis
Source: Front Psychiatry. 2022 May 30;13:847498. doi: 10.3389/fpsyt.2022.847498 (PMC9196636; doi:10.3389/fpsyt.2022.847498)
Supplement: Supplementary file 1 [file Data_Sheet_1.docx]

**Supplementary Table 1.** Linear regression analysis of resilience score in the green group

|  | Univariate analysis | | Multivariate analysis | |
| --- | --- | --- | --- | --- |
| N = 21 | Standardized  Beta | *p* value | Standardized Beta | *p* value |
| PROVE-ACE | 0.635 | 0.119 | 0.451 | 0.315 |
| PROVE-MC | 0.001 | 0.998 | 0.023 | 0.931 |
| Attachment type  (disorganized) | -0.45 | 0.081 | -0.354 | 0.218 |
| Sqrt-AUCg_cortisol_ | 0.058 | 0.829 | -0.007 | 0.979 |
| Age | 0.005 | 0.983 | 0.245 | 0.572 |
| Sex (male) | -0.138 | 0.549 | 0.129 | 0.673 |
| Current Psychiatric medication (Yes) | -0.191 | 0.407 | -0.602 | 0.238 |

PROVE, PROtective and Vulnerable factors battEry questionnaire; ACE, adverse childhood experience; MC, mentalization capacity problem; Sqrt-AUCg_cortisol,_ square root of the value of area under curve with respect to the ground of cortisol awakening response. ^*^: *p* < 0.05, ^**^: *p* < 0.01, ^***^: *p* < 0.001

**Supplementary Table 2.** First sample on awakening of cortisol (S1) by group with PROVE battery, presence of ACE, and resilience group.

| PROVE group | Green (n = 20) | Yellow (n = 30) | | Red (n = 23) | *F* | *p* value |
| --- | --- | --- | --- | --- | --- | --- |
|  | 3.24 (0.72) | 3.26 (0.82) | | 2.69 (1.05) | 1.9 | 0.157 |
| ACE | No (n = 44) | | Yes (n = 29) | | *F* | *p* value |
|  | 3.19 (0.8) | | 2.9 (1.03) | | 3.267 | 0.075 |
| Resilience group | High  (n = 7) | Moderate  (n = 27) | | Low  (n = 39) | *F* | *p* value |
|  | 2.97 (0.6) | 3.31 (0.85) | | 2.93 (0.96) | 1.837 | 0.167 |

PROVE, PROtective and Vulnerable factors battEry questionnaire; ACE, adverse childhood experience
